# Enhanced Emission and Polarization Control of Green GaN-Based Resonant Cavity LEDs with Porous Distributed Bragg Reflectors

**DOI:** 10.1021/acsomega.5c12314

**Published:** 2026-01-30

**Authors:** Yi-Sin Cheng, Safeer Hussain Rather, Kai-Cheng Yu, Chiung-Hua Wang, Chia-Yi Chen, Yen Da Chen, Yu-Cheng Kao, Maria Kiran Kocherla, Der-Yuh Lin, Hongta Yang, Chia-Feng Lin

**Affiliations:** † Department of Materials Science and Engineering, 34916 National Chung Hsing University, 145 Xingda Road, South District, Taichung City 40227, Taiwan; ‡ Department of Electronic Engineering, National Changhua University of Education, No. 2, Shida Road, Changhua City, 500208, Taiwan; § Department of Chemical Engineering, 34916 National Chung Hsing University, 145 Xingda Road, South District, Taichung City 40227, Taiwan

## Abstract

Green InGaN-based resonant-cavity light-emitting diodes (RC-LEDs) with integrated nanoporous (NP) GaN distributed Bragg reflectors (DBRs) were fabricated by using a selective electrochemical (EC) etching technique. The epitaxial structure comprised 25 alternating Si-doped GaN layers, forming a periodic high- and low-refractive-index stack after the EC etching process. The resulting porous-GaN DBR exhibited an effective refractive index of 2.02 and a stopband centered at 566 nm, achieving 98% reflectivity. By integrating a top-3-pairs dielectric mirror, the electroluminescence (EL) spectra showed a narrower full width at half-maximum (fwhm) from 41 nm (nontreated LED) to 12.8 nm (RC-LED). Polarization analysis revealed two spectral peaks at 536.7 and 544.7 nm, with one suppressed by a polarizer. The fwhm varied with the polarization direction, being 5.2 nm at 0° and 6.4 nm at 90°. These results demonstrate that integrating bottom porous GaN and top dielectric DBRs yields a high-reflectivity, low-loss RC structure suitable for high light coupling in fiber interconnection and high-color-purity micro-LED display applications.

## Graphical abstract

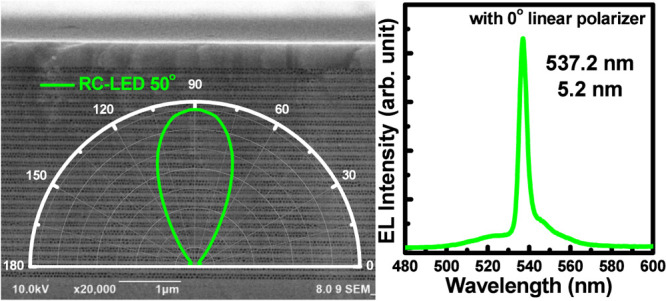

## 1 Introduction

Gallium Nitride (GaN) has gained significant attention as a promising material for various optoelectronic applications, particularly in light-emitting diodes (LEDs).^1–3^ InGaN-based LEDs have been attractive due to the emission spectra covering from near-ultraviolet to green, enabling potential for full color display and widely applied display technology, back lighting, and general illumination areas.^4,5^ However, compared with GaAs- or InP-based vertical-cavity surface-emitting lasers (VCSELs), the development of GaN resonant-cavity light-emitting diodes (RC-LEDs) remains challenging, primarily due to large lattice mismatch with substrates, high defect densities, and difficulties in fabricating lattice-matched distributed Bragg reflectors (DBRs).^6^ The top/bottom dielectric DBR structure^7^ had been reported through the substrate removal process. Recent advances have introduced porous GaN DBRs, fabricated via selective electrochemical (EC) etching of Si-doped GaN layers, as a promising alternative to conventional epitaxial DBRs.^8,9^ These structures achieve high reflectivity with fewer pairs, eliminate lattice mismatch issues, and simplify the fabrication process.^10^ InGaN/GaN multiple quantum wells (MQWs) serve as the active medium, offering tunable emission across visible wavelengths, though challenges such as indium-induced strain and the quantum confined Stark effect (QCSE) continue to limit efficiency in the green spectral range.^11,12^ Directional and polarization-controlled light sources are crucial for emerging applications in AR/VR, optical interconnects, and high-resolution displays. GaN-based light sources with dual-polarization operation can further enable polarization multiplexing in communication systems, improving bandwidth and stability.^13,14^ In addition to spectral flexibility, GaN exhibits high electron mobility, low resistivity, and strong thermal stability, making it suitable for high-power and high-frequency devices.^15^ VCSELs are among the most attractive InGaN-based optoelectronic devices due to their unique advantages, including narrow spectral line width, low threshold current, circular beam emission, and suitability for array integration.^16,17^ These features position GaN-based VCSELs as strong candidates for applications in microprojectors, optical communications, biosensing, AR/VR displays, and high-density 3D imaging.^18,19^

In this work, InGaN-based RC-LEDs with porous GaN DBRs and dielectric DBRs were designed and fabricated. Electrochemical etching for bottom DBRs, ion implantation for current confinement, and dielectric DBR deposition were employed to realize the final devices. A narrow divergent angle and an emission line width were demonstrated in the green RC-LED with a three-pair top dielectric DBR. Optical and spectral properties of the LED with and without the DBR structure are analyzed and discussed in detail.

## 2 Experimental Details

InGaN LED structures were grown on *c*-plane (0001) patterned sapphire substrates using metal–organic chemical vapor deposition (MOCVD). The epitaxial structure consisted of an unintentionally doped GaN buffer layer, a 300 nm Si-doped n-GaN electrical contact layer for the EC etching process (doping concentration: 2.5 × 10^19^ cm^–3^), and 25 pairs of alternating Si-doped GaN layers with an 80 nm-thick high (2.5 × 10^19^ cm^–3^) and 50 nm-thick low (2 × 10^18^ cm^–3^) doping concentration stack structure, an n-type GaN:Si layer, the active region with four pairs of InGaN/GaN MQWs, and a p-type GaN:Mg layer. Initially, laser scribing was performed by using a 355 nm UV laser to define channels spaced 400 μm apart, exposing the conductive n-GaN layer. Indium balls were pressed onto the exposed regions to ensure ohmic contact. The sample served as the anode, while platinum acted as the cathode; both were immersed in a solution of nitric acid, the concentration of which was 0.5 M. A direct-current bias of 8 V was applied under these conditions. The highly Si-doped GaN layers were selectively transformed into nanoporous GaN with a reduced effective refractive index. In contrast, the lightly doped GaN layers remained intact, forming the periodic high- and low-refractive-index stack. The nanoporous GaN (NP-GaN) DBR was fabricated by EC wet etching. The etching process was terminated when the etched length reached 120 μm, resulting in a 240 μm-wide porous mirror region and a 160 μm-wide untreated region for comparative analysis. A 30 nm indium tin oxide (ITO) transparent conductive film was deposited by physical vapor deposition (PVD) at 180 °C, followed by rapid thermal annealing at 500 °C for 30 s to enhance the conductivity and transparency. Current confinement was achieved through nitrogen ion implantation (^14^N^+^, 200 keV). Regions not protected by photoresist became highly resistive, effectively restricting the current flow to the active region and enhancing carrier injection efficiency. Device mesas with dimensions of 200 μm × 100 μm were defined using a laser direct writing system. Inductively coupled plasma (ICP) etching was performed in an ULVAC NE550 system using Cl_2_ gas. The radiofrequency power was set to 200 W at the top electrode and 100 W at the bottom electrode, yielding an etch rate of approximately 3.5 nm/s. The etch depth was confirmed by using scanning electron microscopy (SEM). For ohmic contacts, 20 nm thick Ti and 200 nm thick Al layers were deposited to form p- and n-type electrodes, respectively. Under forward bias operation, holes were injected from the p-GaN side through the ITO layer, while electrons were injected from the n-GaN side through the NP-GaN Bragg mirror, recombining in the MQW active region. Finally, alternating TiO_2_/SiO_2_ DBRs were deposited using an ion plating dual electron gun evaporation system. Three- and 10-pair DBRs were fabricated under oxygen (15 sccm) and argon (10 sccm) ambient conditions. The argon ions enhanced the film density, while substrate heating improved the surface mobility of TiO_2_ and SiO_2_ atoms, resulting in dense, high-quality dielectric mirrors with high reflectivity. The LED structures without EC treatment and top DBR are defined as the nontreated standard LED (ST-LED). LED structures with EC treatment DBR structure are defined as the EC-treated LED (EC-LED). LED structures with EC treatment and a top DBR were designated as RC-LEDs. Cross-sectional micrographs were taken using the field-emission scanning electron microscope (JEOL JIB-4601F) and the transmission electron microscope (JEOL JEM-2100F). The photoluminescence (PL) and electroluminescence (EL) spectra were measured using a monochromator (JOBIN YVON iHR550). A 405 nm diode laser was used for the PL measurement. The polarization EL spectra were measured by rotating the linear polarizer (Thorlabs LPUV050-MP2).

## 3 Results and Discussion

Figure 1a shows the schematic of the RC-LED structure. Figure 1b shows optical microscopy (OM) images of the EC lateral wet etching region to form the porous-GaN DBR structure. The EC wet etching channels were defined at 400 μm spacing, and the lateral EC etching width extended to 120 μm after 1240 s under an 8 V bias, corresponding to a lateral etch rate of ∼5.4 μm/min. The central region in the emission area of the ST-LED that was protected by photoresist remained intact. Figure 1c shows OM images of the ST-LED and RC-LED, side by side for comparison, illustrating the subsequent device fabrication steps, including ITO deposition, aperture patterning by photolithography, and the formation of Ti/Al ohmic contact pads over a 200 μm × 100 μm p–n interface. Finally, dielectric mirrors with varying pair numbers were deposited to complete the RC-LED structure. Multiple device types, including ST, EC, and mirrors with different pair numbers, were fabricated on the same wafer for direct comparison.

**Figure 1:**
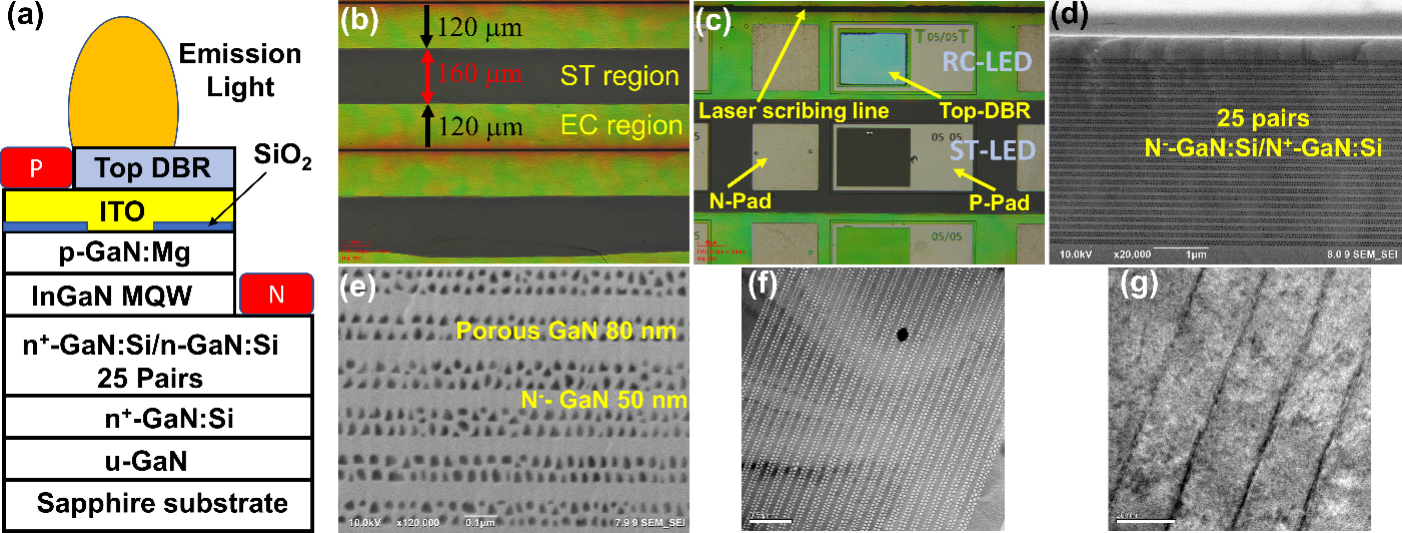
(a) Schematic of the RC-LED structure. (b) OM image of the Bragg mirror at 10× objective. The etching distance is marked. (c) OM image of devices under a 20× objective. (d, e) SEM cross-section images of vertical laser cutting lines at magnifications of (d) 20k (e) 120k for porous DBR structures. (f, g) TEM micrographics of (f) the porous-GaN DBR structure and (g) the MQW active structure.

As shown in Figure 1d,e, SEM analysis was performed on cross sections parallel to the etching channels. The selective etching process converted n^+^-GaN:Si layers into nanoporous GaN, forming an alternating high-/low-refractive-index stack that serves as the porous-GaN DBR structure. The layer sequence from bottom to top includes 3.4 μm-thick 25-pair Bragg mirrors and a 400 nm-thick LED structure. The periodic corrugated structure of the porous Bragg mirror is clearly visible after electrochemical etching. Measurements from SEM images show that the porous GaN layer is 80 nm thick, and the unetched GaN layer is 50 nm thick, as shown in Figure 1d. The effective refractive index was calculated using the following formula:

$$n_{\mathrm{NP-GaN}}=\sqrt{\left(1-\varphi \right){n_{\mathrm{GaN}}}^{2}+\varphi {n_{\mathrm{air}}}^{2}}$$

where φ is the porosity, *n*_GaN_ ≈ 2.46, and *n*_air_ = 1. The porosity of the etched layer was calculated to be 39% using image analysis. The effective refractive index was then estimated to be 2.02.

Figure 1f shows a TEM cross-sectional image in which the periodic porous Bragg mirror, with a thickness of 3.4 μm, is observed as the embedded reflector within the whole epitaxial structure. The InGaN/GaN MQW active region is clearly visible in Figure 1g, consisting of four distinct quantum wells with a 3 nm-thick InGaN well and a 16 nm-thick GaN barrier.

Figure 2a shows reflectance spectra with and without electrochemical etching. The reflectance spectra of the EC-treated LED and nontreated LED structure were measured through the microreflectance measurement including the microscope, tungsten filament lamp, 10× objective lens, HR-4000 spectrometer, and Si substrate as the calibration sample. The measured LED chips were side-by-side chips in the same wafer, in which the epitaxial structure is the same for comparison. Untreated regions exhibited ∼18% reflectivity at 530 nm, while EC-etched porous DBRs reached about 98% with a 47 nm stopband centered at 566 nm. Devices with three dielectric mirror pairs (TiO_2_/SiO_2_) achieved ∼70% reflectivity, and those with 10 pairs exceeded 99% with a 137 nm stopband, demonstrating excellent agreement with theoretical predictions. In Figure 2b, the PL spectra compare the photoluminescence of ST-LED and DBR-LED devices excited by a 405 nm diode laser. The ST-LED emits at 537 nm, while the DBR-LED emission peak was red-shifted from 537 to 543 nm and exhibited 3.2 times higher intensity. The peak wavelength was slightly red-shifted, and the intensity increased in the InGaN active layers, which were affected by the embedded EC-treated porous DBR structure. By forming the embedded DBR in the EC-LED structure, the RC-LED exhibited a higher PL intensity due to the reflected light propagating in the normal direction. The peak wavelength of the RC-LED was slightly red-shifted, attributable to the stopband of the porous DBR structure, due to superposition effects.

**Figure 2:**
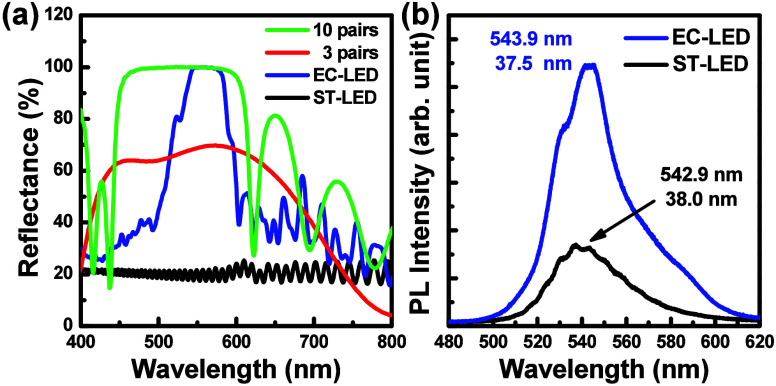
(a) Reflectance spectra of ST-LED, EC-LED, and RC-LEDs with three-pair and 10-pair dielectric DBRs. (b) PL spectra of the ST-LED and RC-LED measured using a 405 nm laser as the excitation laser.

The EL spectra of the ST-LED and the RC-LED structures were studied under injection currents ranging from 0.1 to 5.0 mA, as shown in Figure 3a,b. The EL emission wavelength and full width at half-maximum (fwhm) of the ST-LED were measured as 551.6/40.9 nm at 1 mA and 549.5/41.2 nm at 5 mA, respectively, as shown in Figure 3a,c. For the RC-LED structure, the EL wavelength and the fwhm were observed at 546.7/14.0 nm (1 mA) and 544.3/12.8 nm (5 mA), respectively, as shown in Figure 3b,c. The low EL intensity and broadened line width were observed in the ST-LED due to the quantum-confinement stack effect (QCSE) in the high-indium-content InGaN/GaN MQW active layers. Stable EL emission wavelength and a slight line-width reduction were observed in the RC-LED with a three-pair top dielectric DBR. At 5 mA, the EL emission wavelength was slightly blue-shifted from 546.7 nm (ST-LED) to 544.3 nm (RC-LED) due to reduced QCSE and wavelength pinning at the cavity mode in the RC structure. Compared to the ST-LEDs, the RC-LEDs featuring three top DBR pairs exhibited enhanced EL intensity, with intensity increasing linearly with current because they match the central wavelength of the resonance cavity, resulting in a narrower fwhm of 12.8 nm, as shown in Figure 3c. By forming the top/bottom DBR structures, the high EL intensity and narrow fwhm value were observed in the RC-LED due to the cavity effect between the top dielectric DBR and the bottom porous GaN DBR structures.

**Figure 3:**
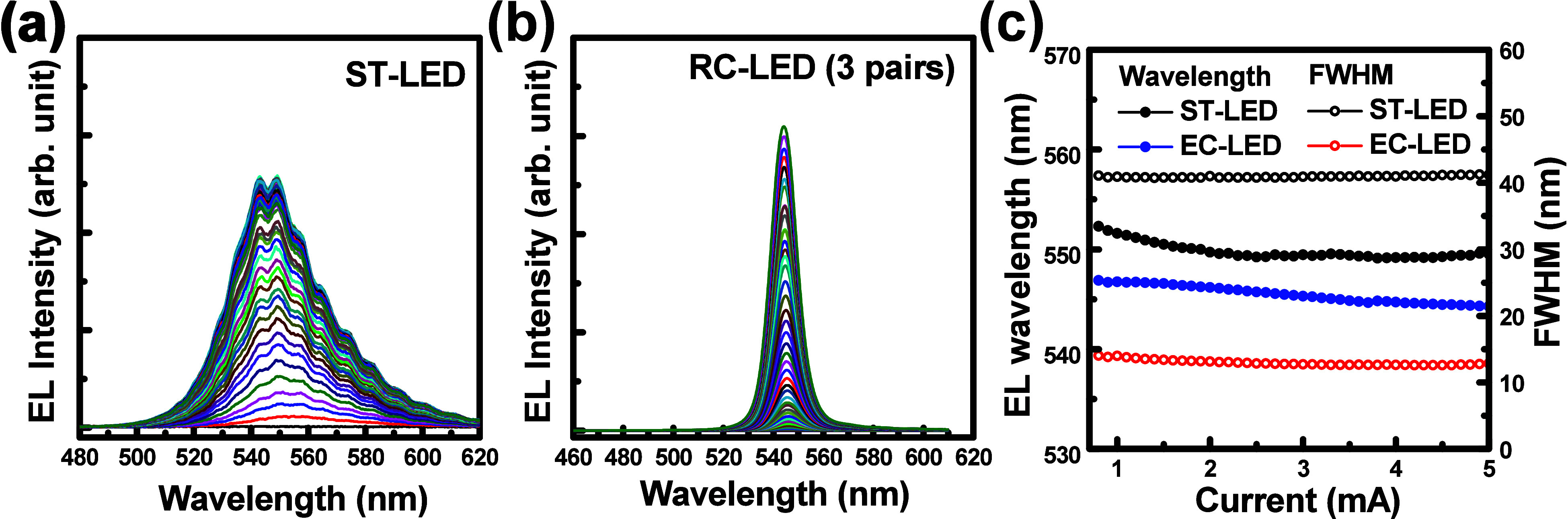
(a, b) EL spectra of (a) the ST-LED and (b) the RC-LED with three-pair top dielectric DBR structure. (c) Analysis of the EL emission wavelength and fwhm in both LED structures.

The angular-dependent EL emission spectra of both LED structures were measured, as shown in Figure 4a,b, using a 200 mm fiber without an optical lens and a 5 cm distance between the LED and the fiber. When the fiber was moved to the normal direction, the EL emission spectra of both LED structures were measured as the injection current was varied. Low EL intensity of the ST-LED was observed compared with the EC-LED structure in the normal direction. Angular-resolved EL spectra at a constant 5.0 mA were measured at normal incidence from 0° to 180°, further distinguishing the device emission characteristics. High density of the interference curves was observed in the ST-LED, as shown in Figure 4a. That is due to the emission light from the MQW active layer exhibiting interference between the top air/ITO-GaN:Mg and bottom GaN/sapphire interfaces. As shown in Figure 4a,c, strong side peaks were present in the ST-LED due to the light scattering from the patterned sapphire substrate. In the RC-LED structure, a narrow emission pattern and only one interference line were observed, as shown in Figure 4b. In Figure 4c, the divergent angle is defined as the detected angle between two angles of the half EL emission intensities compared with the normal direction EL emission intensity. The divergent angles, measured at 50% peak intensity, were 128° for the ST-LED and 50° for the RC-LED with a three-pair top DBR structure. The divergent angle of the LED structure can be reduced by adding top and bottom DBRs to form a resonant cavity.

**Figure 4:**
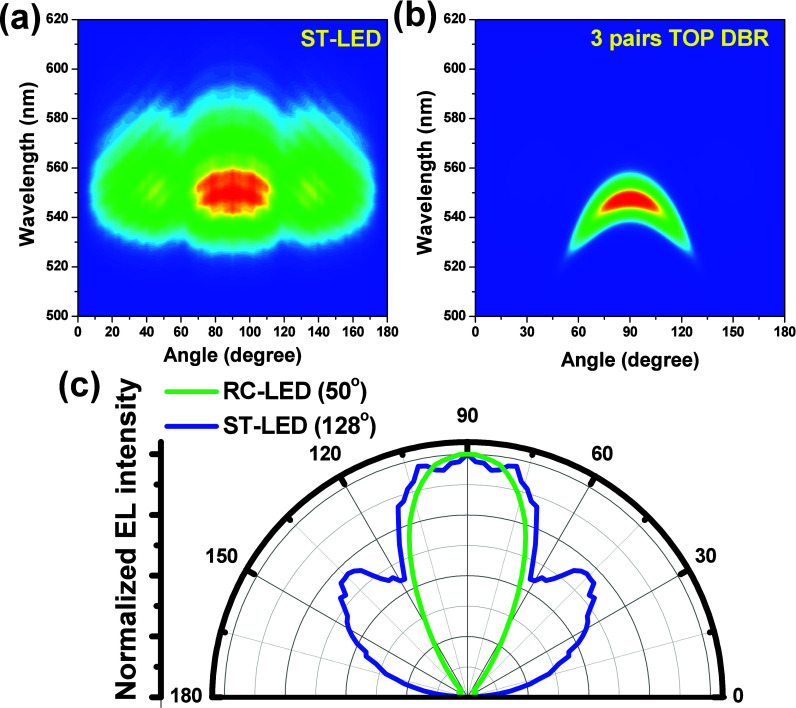
(a, b) Normalized far-field emission spectra of (a) ST-LED and (b) RC-LED with three-pair top dielectric DBR structure resolved at 5 mA operation current. (c) Far-field intensity distribution curves measured at 5.0 mA.

The EL spectra of the RC-LED containing the 10-pair top dielectric DBR were measured under varying injection currents. As shown in Figure 5a, the EL peak wavelengths and the fwhm were found to be 537.2 and 3.5, 544.9 and 4.6, and 536.9 and 39.9 nm, respectively, at 5 mA in the RC-LED structure without the linear polarizer, demonstrating strong spectral confinement. The broadened EL peak at 536.9 nm with 39.9 nm line width came from the InGaN MQW active layer. Using the linear polarizer, the EL peak wavelengths of the RC-LEDs were measured at 537.2 nm with a 0° linear polarizer and 544.9 nm with a 90° linear polarizer, respectively, at a 5 mA injection current, as shown in Figure 5b,c. The polarization effect was attributed to the anisotropic pipe structure of the porous DBR, which induced separation of the cavity modes due to the pipe–GaN/GaN refractive index contrast. The pipe structure is perpendicular to the laser scribing (LS) line, and 0° is defined when the linear polarizer is parallel to the pipe direction (perpendicular to the LS line). The highest EL emission intensity was observed at 0°, whereas the lowest was at 90°. The multimodal spectral features observed are due to the polarization characteristics of the bottom Bragg mirror. This device exhibits strong angle-dependent emission consistent with resonant cavity phase-matching conditions, supporting an enhanced directional emission. The bottom porous GaN DBR has an anisotropic pipe structure with different cavity modes. The tubular geometry of the porous Bragg reflector creates anisotropic refractive indices along and across the pores, leading to polarization-dependent EL spectra. The ratio of the EL intensities of the 537.2 and 544.9 nm peaks obtained by rotating the linear polarizer was 1.8 at 1.6 mA and 1.6 at 5 mA. The EL peak intensity of the RC-LED with and without a linear polarizer was measured, as shown in Figure 5d. The RC-LED without the linear polarizer had an EL intensity higher than that with the polarizer, indicating that the polarizer absorbed light. The EL emission intensity of the RC-LED structure was row-over due to Joule heating and low efficiency at high injection currents. As shown in Figure 5e,f, with a 0° linear polarizer, the peak wavelength and fwhm values were observed at 539.7 and 4.7 nm at 0.5 mA and at 537.2 and 5.2 nm at 5 mA, respectively, whereas with a 90° linear polarizer, the peak wavelength and fwhm values were observed at 546.8 and 5.7 nm at 0.5 mA and at 544.9 and 6.5 nm at 5 mA, respectively. The fwhm values increased slightly with increased injection current due to thermal broadening. The polarization-induced wavelength shift was about 7.7 nm in this reported RC-LED with top isotropic dielectric DBR and bottom anisotropic porous GaN DBR structures. These two emission peaks in one micro-LED chip have the potential to be used for short-distance optical communication and double the rate (Gbps) in an AI data center to replace the Cu line transition.

**Figure 5:**
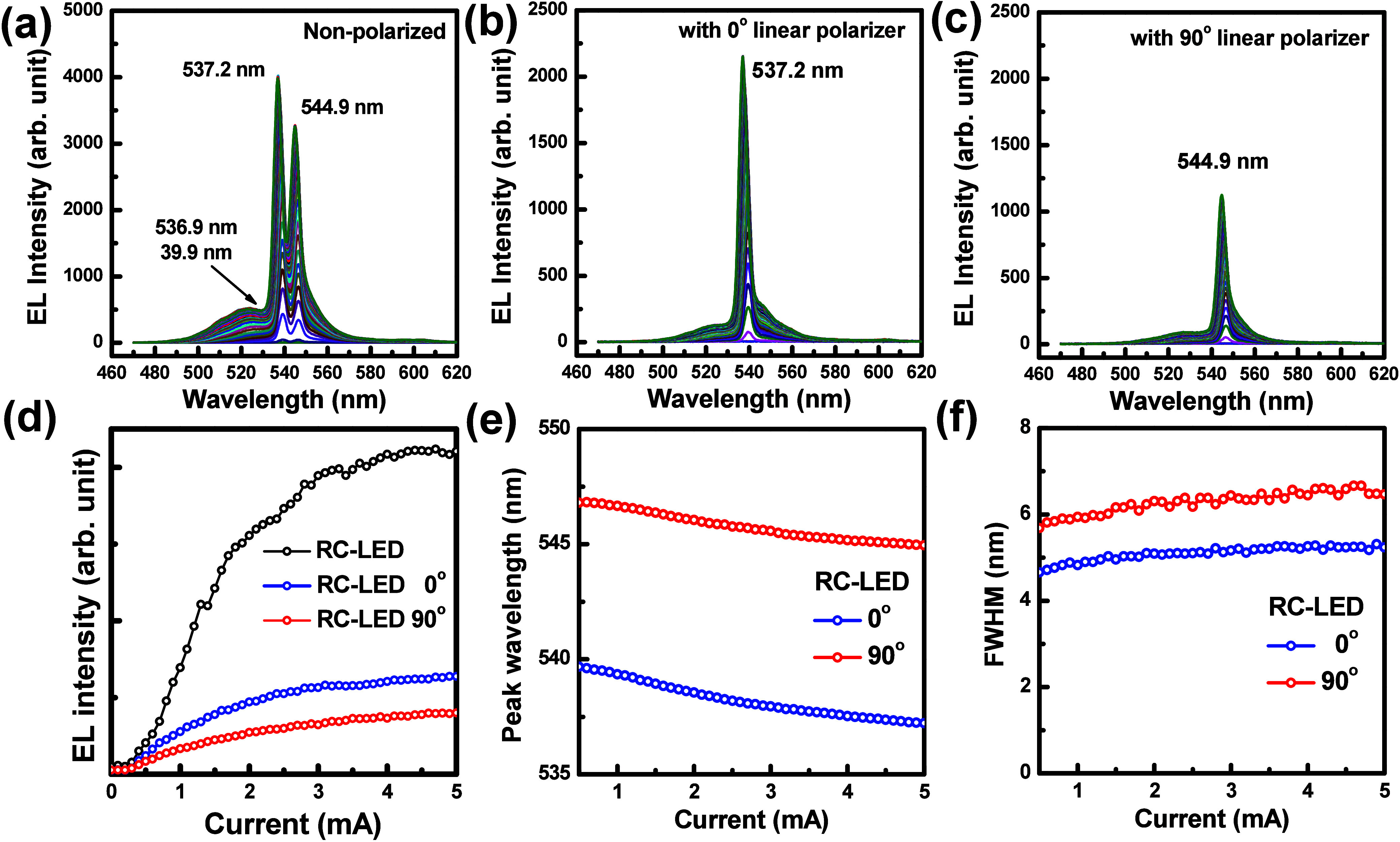
(a–c) EL spectra (a) without polarizer, (b) with a linear polarizer at 0°, and (c) with a linear polarizer at 90° measured with varying injection current. (d) Trends of the EL intensity, (e) peak EL wavelength, and (f) fwhm analyzed by varying the injection current.

## 4 Conclusion

The green RC-LEDs demonstrated incorporating an electrochemically formed nanoporous-GaN DBR as an embedded reflector and a TiO_2_/SiO_2_ dielectric mirror as a top cavity mirror. The selective EC process enabled precise lateral formation of high-contrast refractive-index periodic structures. Optical characterizations confirmed high reflectivity, enhanced luminescence intensity, and spectral narrowing due to strong cavity resonance in the InGaN RC-LED. The RC-LED exhibited suppressed wavelength shift and improved carrier confinement compared to conventional ST-LEDs. This approach eliminates lattice-mismatch and stress issues associated with conventional epitaxial DBRs while enabling monolithic, low-cost cavity fabrication. Controllable porosity and refractive-index contrast in the porous-GaN/GaN stack structure open a new path for engineering integrated photonic devices with tailored optical confinement and resonance properties. The future applications of this technology extend beyond general lighting, particularly toward high-color-purity micro-LED displays, optical interconnects for data centers, and cavity-enhanced detectors.

## References

[1] Nakamura S. (1998). The roles
of structural imperfections in InGaN-based blue light-emitting diodes
and laser diodes. Science.

[2] Yulianto N., Kadja G. T. M., Bornemann S., Gahlawat S., Majid N., Triyana K., Abdi F. F., Wasisto H. S., Waag A. (2021). Ultrashort
pulse laser lift-off processing of InGaN/GaN light-emitting diode
chips. ACS Appl. Electron. Mater..

[3] Zhang G., Guo X., Ren F.-F., Li Y., Liu B., Ye J., Ge H., Xie Z., Zhang R., Tan H. H., Jagadish C. (2016). High-brightness
polarized green InGaN/GaN light-emitting diode structure with Al-coated
p-GaN grating. ACS Photonics.

[4] Li H., Kang J., Li P., Ma J., Wang H., Liang M., Li Z., Li J., Yi X., Wang G. (2013). Enhanced performance of GaN-based light-emitting diodes
with a low-temperature
p-GaN hole injection layer. Appl. Phys. Lett..

[5] Li P., Li H., Wang L., Yi X., Wang G. (2015). High quantum efficiency
and low droop of 400-nm InGaN near-ultraviolet light-emitting diodes
through suppressed leakage current. IEEE J.
Quantum Electron..

[6] Alam S. N., Zubialevich V. Z., Ghafary B., Parbrook P. J. (2020). Bandgap
and refractive
index estimates of InAlN and related nitrides across their full composition
ranges. Sci. Rep..

[7] Chen, H. ; Li, Z. ; Lei, M. ; Genc, M. ; Meng, L. ; Roycroft, B. ; Chen, W. ; Hu, X. ; Corbett, B. GaN-based Resonant Cavity LEDs Fabricated by Photo-Electrochemical Etching and Micro-Transfer Printing. arXiv (Physics.Optics), October 21, 2025, 2510.18507, ver. 1. https://arxiv.org/abs/2510.18507.

[8] Zhang C., Park S. H., Chen D., Lin D.-W., Xiong W., Kuo H.-C., Lin C.-F., Cao H., Han J. (2015). Mesoporous
GaN for photonic engineering — highly reflective GaN mirrors
as an example. ACS Photonics.

[9] Zhu T., Liu Y., Ding T., Fu W. Y., Jarman J., Ren C. X., Kumar R. V., Oliver R. A. (2017). Wafer-scale fabrication of non-polar
mesoporous GaN distributed Bragg reflectors via electrochemical porosification. Sci. Rep..

[10] Griffin P. H., Patel K. M., Zhu T., Langford R. M., Kamboj V. S., Ritchie D. A., Oliver R. A. (2020). The relationship between the three-dimensional
structure of porous GaN distributed Bragg reflectors and their birefringence. J. Appl. Phys..

[11] Mei Y., Xu R. B., Ying L.-Y., Liu J. P., Zheng Z. W., Long H., Zhang B. P. (2019). Room temperature
continuous-wave
lasing of GaN-based green vertical-cavity surface-emitting lasers. Proc. SPIE.

[12] Hsieh D. H., Tzou A. J., Kao T. S., Lai F. I., Lin D. W., Lin B. C., Lu T. C., Lai W. C., Chen C. H., Kuo H. C. (2015). Improved carrier injection in GaN-based VCSEL via AlGaN/GaN
multiple quantum barrier electron blocking layer. Opt. Express.

[13] Wu C. J., Chen Y. Y., Wang C. J., Shiu G. Y., Huang C. H., Liu H. J., Chen H., Lin Y. S., Lin C. F., Han J. (2020). Anisotropic properties of pipe-GaN distributed Bragg reflectors. Nanoscale Adv..

[14] Ke Y., Wang C.-J., Shiu G.-Y., Chen Y.-Y., Lin Y.-S., Chen H., Han J., Lin C.-F. (2022). Polarization properties
of InGaN vertical-cavity surface-emitting laser with pipe distributed
Bragg reflector. IEEE Trans. Electron Devices.

[15] Meneghini M., De Santi C., Abid I., Buffolo M., Cioni M., Khadar R. A., Nela L., Zagni N., Chini A., Medjdoub F., Meneghesso G., Verzellesi G., Zanoni E., Matioli E. (2021). GaN-based power devices: Physics,
reliability, and perspectives. J. Appl. Phys..

[16] Yang T., Chen Y.-H., Wang Y.-C., Ou W., Ying L.-Y., Mei Y., Tian A.-Q., Liu J.-P., Guo H.-C., Zhang B.-P. (2023). Green vertical-cavity
surface-emitting lasers based on InGaN quantum dots and short cavity. Nano-Micro Lett..

[17] Hamaguchi T., Tanaka M., Nakajima H. (2019). A review on the latest
progress of
visible GaN-based VCSELs with lateral confinement by curved dielectric
DBR reflector and boron ion implantation. Jpn.
J. Appl. Phys..

[18] Yu H.-C., Zheng Z.-W., Mei Y., Xu R.-B., Liu J.-P., Yang H., Zhang B.-P., Lu T.-C., Kuo H.-C. (2018). Progress
and prospects of GaN-based VCSEL from near UV to green emission. Prog. Quantum Electron..

[19] Elafandy R. T., Kang J.-H., Mi C., Kim T. K., Kwak J. S., Han J. (2021). Study and application
of birefringent nanoporous GaN in the polarization
control of blue vertical-cavity surface-emitting lasers. ACS Photonics.

